# Notes and News

**Published:** 1890-04-05

**Authors:** 


### Notes and News.

Collected and Communicated.

One festive contributor wants to know whether the correspondence on the question "Should Acting Members of Medical Staffs be allowed to sit upon Boards of Management?" might be followed by a similar series on the similar question, "Should Boards of Management be allowed to sit upon Acting Members of the Medical Staff?" We think not, but our columns are open.

The South Charitable Infirmary at Cork may well be proud of having reduced its bill for stimulants from £153 in 1883, to £69 last year. The hospital treated 938 in-patients last year; it is economically managed, but needs more annual subscribers, or it will have to reduce its be ls. Surely there are plenty of generous-hearted people in Cork to support such a deserving institution as this?

The bulky report of Edinburgh Royal Infirmary largely consists of list of contributors. It is a large institution and needs wide support and gets it; the ordinary expenditure was £35,917 last year. Over 8,000 patients were treated in the Infirmary last year, and 990 were treated at the Convalescent House at Corstorphine. Legacies amounting to £30,455 were received.

In connexion with the Children's Hospital at Philadelphia is a Croup Ward, specially fitted with tiled walls, extra steam heating, and other appliances to secure warmth and moisture. The ward is entirely isolated; children suffering from diphtheritic disease were admitted, and the success of the tracheotomies performed was above the average. But in the report of this hospital just come to hand, we find the Board of Health refuses to permit diphtheria cases to be treated at the hospital any more, and henceforth such cases must be treated in their own homes. This order requires explanation.

At the late meeting of the Metropolitan Asylums Board, special reference was made to the Ambulance Service, and the committee reported the fact that during the year 25 persons, certified to be suffering from small-pox, were removed from their homes, and of these no fewer than 20 were found not to have the disease. During last year 8,908 cases of fever were removed, as against 10,516 the year before; and in nine years the ambulance has conveyed 69,786 fever and small-pox patients between homes and hospitals.

Sir Morell Mackenzie writes in the *Fortnightly Review* on the Reform of the College of Surgeons. In *Time* there is an article on the Black Plague, and in the *National Review* an article on Rabies and Muzzling, by A. Shadwell, M.R.C.P.

The annual report of the Children's Sanatorium at Southsea, mentions with regret the death of Dr. Harvey, in whose memory they have named one of the wards. The first free cot has been endowed by Miss Geddes. The medical officers acknowledge the help they have received from the present matron and the staff of nurses. Dr. Blumberg, the founder of the institution, has resigned his post as principal physician, to the regret of all concerned.

The hours of asylum attendants is likely to be a burning question before the County Council soon. These men and women are on duty as a rule from six a.m. to eight p.m., and once a-week till ten p.m. They are off duty one half-day a-month and one whole day. These hours may vary in different institutions, but it is evident that now a higher class of workers are becoming attendants, more consideration must be shown them. The local papers are just now full of complaints of Banstead Asylum hours, and we should be glad to see the whole subject of asylum attendants brought more before the public.

Mr. Augustus Prevost, treasurer and chairman, presided at the annual meeting of the University College Hospital on Friday. The report, which was adopted on the motion of the chairman, stated that the debt at the close of the previous year was £18,419. Fortunately, owing to the falling in of a legacy from the late Mr. Goldenberg, the committee were enabled to clear off nearly £11,000 of that amount. The "Hospital re-building Fund" amounted to £11,500, but it would be necessary to raise £30,000 before the work, which is urgently needed, could be undertaken. There was a falling off in the donations by £609, and in subscriptions by £111, and the total receipts were so far insufficient that the year closed with an indebtedness of £10,000. Stock had been sold out to the value of £1,111 11s. 9d. to meet pressing claims. The reliable income of the Hospital is only £6,000, whereas the expenditure exceeds £20,000, and the committee urgently appeal for increased support in donations and subscriptions.

Bristol Children's Hospital reports that it treated 753 small patients last year, the largest number recorded since the opening of the hospital. The measels ward has been specially well filled. The financial statement shows that the subscriptions amounted to £1,109, the donations to £736 18s. 6d., and the collections at churches and chapels, including church parades of various friendly societies, to £384 2s. This is not an increase in proportion to the increase of patients, and there is over £400 due to the treasurer. Speaking at the annual meeting the Ven. Archdeacon Norris said he thought the institution needed no special pleading for. The beauty of the building, its cloistered garden, and still more the interior, as all the wards seemed to remind them of their fragrance, and sweetness, and cleanliness—all these things pleaded for the funds of the institution; and the report, one of the most business-like he had heard, was a strong plea in itself. He was amazed at the small amount of administration charges compared with the work done. It seemed to him that if they divided the one with the other they would find that this institution was most economically carried on. Compared with other institutions, the economy in the management was very striking indeed. Mr. Whitwill, the energetic president of the institution, said he gave to the matron the credit of the economical management of the institution.

The following vacancies are announced this week, the amount of the salary in each case being given after the name of the institution:—

**Resident Medical Officers.**

Bethlem Hospital, Two Clinical Assistants—Board, etc.
Brompton Consumption Hospital, House Physicians.
Bath General Hospital, Medical Officer—£100, board, etc.
Kent County Asylum, Senior Assistant Medical Officer—£250, rooms, etc.
Kent County Asylum, Second Assistant Medical Officer—£180, rooms, etc.
Kent County Asylum, Third Assistant Medical Officer—£150, rooms, etc.
Shadwell Hospital for Children, House Physician—Board, etc.

**Non-Resident Medical Officers.**

Owen's College, Manchester, Senior Demonstrator in Physiology —£150.
Stockton-on-Tees Hospital, House Surgeon—£200.
Victoria Park Chest Hospital, Pathologist—£100.
Royal College of Physicians, Milroy Lecturer.
King's College, Professor of Psychological Medicine.
The Hospital for Women, Clinical Assistants.
University of Edinburgh, Additional Examiner—£90.
Gloucester Eye Institution, Assistant Physician.
Royal Westminster Ophthalmic Hospital, Surgeon.

A crowded meeting of East-end delegates was held on Saturday evening at the London Hospital, in connexion with the coming great demonstration of friendly and trade societies on Saturday, May 10th, in aid of the Morley House Convalescent Home, St. Margaret's Bay, Dover. The societies in the four divisions of London, with their bands and banners, will meet in separate sections, and march to the Victoria Embankment, and thence to the Albert Hall, where a musical entertainment will be given, and will be attended by the Lord Mayor and Sheriffs.

A report to make philosophers weep is that of the Dental Hospital of London, which last year treated 54,630 cases of toothache in some form or another. What an awful vision of suffering does this call up! Fancy 54,000 people with the toothache, and the thought is enough to drive you mad. Still sadder is it to see that this number is an increase of 3,000 on the number treated in 1888. Evidently our teeth are growing steadily worse, and our only consolation is that our knowledge of anæsthetics is growing steadily better, and that dental science can provide us with molars which never ache. Let us encourage dental science then, and help our poorer friends who have toothache by subscribing to the Dental Hospital in Leicester Square which sadly needs enlarging.

Easter offerings have long been popular in this country, and many people desire to bestow their gifts wisely and well. We commend to their attention the Alexandra Hospital for Hip Disease, 18, Queen Square, Bloomsbury, W.C., which is sadly in want of help at the moment. We had to visit this hospital unexpectedly one evening last week, and no one connected with the establishment was aware of our intention. There were sixty children of all ages in the wards, and a more pathetic sight, on account of the nature of their ailment, is not to be met with in the whole of London. Everything was in the most perfect order, the wards were airy and perfectly kept, the children—though compelled to lie on their backs—were quiet, comfortable, and happy, and the staff were alert and active. We wish that all who take an interest in children, and especially those who have long purses and generous hearts, may be induced to visit this institution during the next few weeks, and then its coffers will be speedily replenished. The Alexandra Hospital is worthy of the honoured name it bears, but we could wish its committee were more alive to the importance of making the claims of this excellent hospital more generally known. Miss Moore, the lady superintendent, is one of the ablest and most devoted of hospital matrons.

Cheltenham General Hospital held its annual meeting on March 21st, General Williams presiding. The hospital is well administered and well supported as a rule, but unfortunately last Hospital Sunday was extremely wet, and the collections were small. Over 700 in-patients were treated last year, and over 3,000 out-patients. The ordinary expenditure was £4,392.

The annual report of Leicester Infirmary is a bulky but interesting volume, and contains leaflets giving particulars of the Children's Hospital and the Sutton Charity. In connection with the Infirmary is the Fever House of Recovery, which is a particularly useful institution; there is also an eye department which had 177 in-patients last year and 1,475 out-patients. The average cost of each patient in the infirmary is £3 0s. 6½d., and the total expenditure for the year 1889 was £9,275, being £150 less than the year before.

The following announcement appeared in the list of deaths on Saturday last: "27th inst., at the London Hospital, of typhoid fever, taken in the discharge of his duties as house physician, R. Gerard Wilde, M.A., M.B., B.C., of Clare College, Cambridge, elder son of the Rev. R. Wilde, H.M. Inspector of Schools, aged 27." Mr. Wilde is deeply regretted by all who knew him; his unfailing kindness had won all hearts to him, and his loss will be severely felt by everyone at the London Hospital. As only one specimen of Mr. Wilde's goodness, it may be mentioned that last summer, when he went for a short holiday, he took with him a small patient from the children's ward, who was sadly in need of change of air, and was unable to gain admittance to a convalescent home.

The report of the Nottingham Samaritan Hospital for Women say, that while the subscriptions probably on account of the bad or indifferent trade, have fallen off, the donations have increased on the previous year. It is stated, however, that if the progress of last year continued, next year they would be in the cheerful position of having a hospital without an adverse balance. The liabilities at the end of 1888 were £681 5s. 5d., and this had been reduced on December 31, 1889, by the sum of £301 4s. 8d., of which £270 consisted of the net proceeds of a bazaar, which took place at the close of November. Since the opening of the hospital in 1885, 540 in-patients have been treated, and 8,422 out-patients. The report of the medical officers testifies to the unvarying devotion to her duties of the late matron, Miss Nicholas, "whose recent resignation will involve a loss to the institution which it will be difficult to replace."

How is it the Hospital for Sick Children, in Great Ormond Street, always manages to appeal so forcibly to our sympathies? Many a foundation-stone have we seen laid, and many a hospital function have we attended, but none ever resembled the touching scene at the Hospital for Sick Children last week. There was no Guard of Honour, there was no band, there was no marquee, there were no Royal visitors. Some rude tarpaulins were thrown over the space immediately round the great stone, and in a rude gallery was a small harmonium surrounded by a choir of nurses in uniform. The visitors, only numbering about 100, lent against the rough brick walls, and a little patient on crutches limped forward and spread the mortar and laid the foundation-stone. And then the Rev. Dacre Craven said a few prayers and a hymn was was sung, and mingling with the hymn which rose on the soft spring air, was the clink of the masons' trowels as they continued their work on the rapidly growing walls. To whoever belongs the pretty and pathetic idea of letting a little patient lay the foundation stone, it is worthy of imitation; and the new wing of the Great Ormond Street will not lose in interest because of the simple ceremony which attended its foundation.

